# Defining Optimal Cut-Points for Cardiorespiratory Fitness Associated With Overweight/Obesity in Children: A School-Based Study

**DOI:** 10.3389/fphys.2022.784787

**Published:** 2022-03-10

**Authors:** Mario Kasović, Lovro Štefan, Vilko Petrić, Vesna Štemberger, Iva Blažević

**Affiliations:** ^1^Department of General and Applied Kinesiology, Faculty of Kinesiology, University of Zagreb, Zagreb, Croatia; ^2^Department of Sport Motorics and Methodology in Kinanthropology, Faculty of Sports Studies, Masaryk University, Brno, Czechia; ^3^Recruitment and Examination (RECETOX), Faculty of Science, Masaryk University, Brno, Czechia; ^4^Department of Educational Studies, Faculty of Teacher Education, University of Rijeka, Rijeka, Croatia; ^5^Department of Primary Teacher Education, Faculty of Education, University of Ljubljana, Ljubljana, Slovenia; ^6^Department of Primary Teacher Education, Faculty of Educational Science, University of Pula, Pula, Croatia

**Keywords:** maximal oxygen uptake, anthropometric indices, primary-school students, receiver operating curve, diagnostics

## Abstract

The main purpose of the study was to define optimal criterion-referenced cut-points for cardiorespiratory fitness (CRF) associated with overweight/obesity. In this cross-sectional study, participants were 1,612 children aged 7–14 years (mean age ± SD = 9.7 ± 2.4 years; 52.5% girls). CRF was assessed by the Maximal multistage 20-m shuttle run test, from which maximal oxygen uptake (VO_2_max) was estimated. Anthropometric indices included body-mass index (BMI), waist circumference (WC), and waist-to-height ratio (WHtR). Receiver operating characteristic (ROC) curves were performed to determine cut-off points. In boys, the optimal cut-off points of CRF in defining overweight/obesity for BMI, WC, and WHtR were 44.6, 46.4, and 46.9 mlO_2_/kg/min. The areas under the curves (AUC) were 0.83 (95% CI 0.78–0.88, *p* < 0.001), 0.77 (95% CI 0.71–0.83, *p* < 0.001), and 0.90 (95% CI 0.86–0.93, *p* < 0.001). In girls, the optimal cut-off points were 41.0, 40.8, and 40.7 mlO_2_/kg/min for BMI, WC, and WHtR, with the AUCs of 0.86 (95% CI 0.82–0.90, *p* < 0.001), 0.83 (95% CI 0.79–0.88), and 0.88 (95% CI 0.84–0.93, *p* < 0.001). In conclusion, our newly developed cut-off points for CRF assessed by the Maximal multistage 20-m shuttle run test may adequately detect primary school-aged boys and girls with general and abdominal obesity.

## Introduction

Childhood overweight and obesity have become a major public health concern worldwide [NCD Risk Factor Collaboration (NCD-RisC), 2017], and rising trends have been observed in both developed (Gomes et al., 2014) and less developed countries (Gupta et al., 2012). Estimates suggest that the prevalence of overweight and obesity is between 20 and 45% in European children (Garrido-Miguel et al., 2019). According to the World Health Organization (2017), the rising proportion of overweight and obese children continued to increase in most European countries, with the highest prevalence in southern European and Mediterranean countries. Comparing to the regional estimate, the prevalence of overweight and obesity in Croatian children is higher and ranges between 30 and 45% (Milanović et al., 2020). Unfortunately, being overweight or obese in childhood leads to health-related consequences later in life, including premature mortality (Lindberg et al., 2020), higher prevalence of cardiovascular and metabolic diseases (Reilly and Kelly, 2011), and lower levels of cardiorespiratory fitness (CRF) during adolescence (Tuan et al., 2018).

To measure overweight/obesity, several anthropometric indices have been proposed and validated (Santos et al., 2012), including body-mass index (BMI), waist circumference (WC), and waist-to-height ratio (WHtR). Although the BMI has often been used as an indicator of general adiposity (Ogden et al., 2002), it cannot accurately discriminate between the body fat and lean body mass (Brambilla et al., 2006). On the other hand, WC and WHtR have been proposed to be better anthropometric indicators of central obesity and stronger predictors of mortality (Ashwell et al., 2014; Cerhan et al., 2014). Because of low-cost and simple to measure characteristics, these indicators of general and abdominal obesity are often recommended to be used in epidemiological studies.

Cardiorespiratory fitness is an important indicator of overall health in children (Lang et al., 2018). It has been reported that lower levels of CRF in late adolescence may increase the risk of all-cause mortality in adulthood (Högström et al., 2014). Both CRF and excess fat have been associated with some cardiometabolic risk factors (Díez-Fernández et al., 2014). However, it is less known to which extent does the adjustment of adiposity modifies the association between CRF and cardiometabolic risk at the population level (Silva et al., 2018). A great challenge of relating CRF with health indicators is to define optimal cut-off points for CRF capable of adequately distinguishing between healthy and unhealthy individuals (Silva et al., 2016). A most recent systematic review has found 10 studies defining criterion-referenced standards for CRF (Lang et al., 2019). The shortcomings in these studies included the region-specific and relatively small samples of children, which may not be representative to the whole population. Also, optimal cut-off points for CRF to screen for overweight/obesity has not yet been identified among Croatian children. By obtaining such findings, it would be possible to compare and verify different cut-off points in different populations, and to establish international criterion-referenced standards.

Therefore, the main purpose of the study was to define optimal criterion-referenced cut-points for CRF associated with overweight/obesity.

## Materials and Methods

### Study Participants, Design, and Procedure

In this cross-sectional study, we recruited children aged 7–14 years from the city of Zagreb, the capital city of Croatia. A detailed description of recruitment and procedure has been published previously (Kasović et al., 2021a). In brief, a random sampling approach was used to select primary schools, with each school having equal probability of selection. Before the study began, we had contacted principles from 16 schools to take part in the study. After the initial screening, 12 schools agreed to participate. At the second stage, we randomly selected one class presenting one age group within each school, which gave a total of 1,950 students. The inclusion criteria were: (1) being healthy without physical or mental problems diagnosed by the doctor, (2) regularly attending physical education classes, and (3) those who had height, weight, and waist circumference measured and completed the Maximal multistage 20-m shuttle run test. Of these, 338 did not have a measure of the Maximal multistage 20-m shuttle run test or were absent from school during the testing day. Analyses were performed on 1,612 school aged children (response rate = 82.7%, 52.5% girls). Testing procedures were standardized in order to minimize the effects of environmental factors and to avoid fatigue (Venckunas et al., 2018). CRF was assessed from September to October and all schools were evaluated at the same time. Prior the testing, each teacher was instructed about the testing methodology to standardize the procedure across all schools and classes. During the testing, children wore light T-shirt, shorts, and training shoes. All procedures performed in this study were anonymous and were conducted according to Declaration of Helsinki. The study was approved by the Faculty of Kinesiology, University of Zagreb, Croatia. The informed consent voluntarily was signed by the participants, participants’ parents or their guardians.

### Anthropometric Indices

Height and weight were objectively measured using stadiometer and digital scale with a precision of 0.1 cm and 0.1 kg. BMI was calculated by dividing weight in kg with height in m^2^ [weight (kg)/height (m)^2^]. To define overweight/obese according to BMI, the 85th BMI for age, the Centers for Disease Control and Prevention (CDC) reference percentiles were used, as done in previous studies (Ogden et al., 2002; Pojskić and Eslami, 2018). WC was measured for each participant while standing still. We used anthropometric tape placed horizontally midway between the lower rib margin and the iliac crest at the end of normal expiration (Alberti et al., 2006). WHtR was calculated as WC (in cm) divided by the height (in cm). WC and WHtR were used as indicators of abdominal fat, with values above 85th percentile (Taylor et al., 2000) and a cut off of 0.5 for WHtR (McCarthy and Ashwell, 2006) have been used to identify abdominal obesity in children.

### Cardiorespiratory Fitness

The Maximal multistage 20-m shuttle run test was used to assess the level of CRF (Ortega et al., 2008, 2011; Silva et al., 2018). Detailed information about the testing procedure is described elsewhere (Ortega et al., 2008, 2011; Silva et al., 2018). In brief, all participants were instructed to run back and forth between two parallel lines, 20 m apart, following the pace of an audio signal that began at a speed of 8.5 km/h and increased by 0.5 km/h at 1-min intervals. The measurement was undertaken indoors as the primary testing location (Silva et al., 2018). The final score was written as the number of stages completed during every-minute increasing pace of 20-m shuttle run test. Maximal oxygen uptake (VO_2_max) was estimated using Ruiz et al. (2008) equations. An artificial neural network-based equation to estimate VO_2_max includes sex (boys = 1; girls = 2), age (year), weight (kg), height (cm), and The Maximal multistage 20-m shuttle run test stage (Ruiz et al., 2008). Previous study has shown that the artificial neural network-based equation is significantly correlated with the measured VO_2_max (*r* = 0.96, *p* < 0.001).

### Statistical Analyses

Basic descriptive statistics of the study participants are presented as means and SD. Sex differences were examined with Student *t*-test for independent samples. The effect size of the comparisons between the sexes was calculated using Cohen’s *D* effect size (ES). ES was classified as trivial (<0.2), small (0.2–0.6), moderate (0.6–1.2), large (1.2–2.0), very large (>2.0), and extremely large (>4.0; Hopkins et al., 2009). To determine the discriminatory ability of CRF to predict overweight/obesity for each anthropometric index (BMI, WC, and WHtR), we used receiver operating characteristics (ROC) curves quantified by the area under the curve (AUC). ROC curves analyses are specialized for demonstrating discriminatory power of a certain diagnostic test, where the curve of the test skews closer to the upper left corner (Hanley and McNeil, 1982). The AUC represents the diagnostic power of a test. Rice and Harris (2005) proposed a classification of the AUC as follows: (1) 0.55–0.62 (small), (2) 0.63–0.71 (moderate), and (3) >0.71 (large). Sensitivity and specificity characteristics were calculated and presented as percentages (%). Kappa coefficient (κ) was used to calculate the correlations between the newly established cut-off points for CRF and overweight/obesity. Finally, a set of logistic regression analyses with odds ratios (OR) and 95% CI were performed to determine the classification assessment of the association between low levels of CRF and overweight/obesity. Sex-specific analyses were performed, since there were significant differences between boys and girls in WC, WHtR, and CRF. Two-sided values of *p* were used, and significance was set at *α* < 0.05. All the analyses were calculated in Statistical Packages for Social Sciences v.23 (SPSS, Chicago, IL, United States).

## Results

Basic descriptive statistics of the study participants are presented in Table 1. Boys were taller, heavier, and had significantly higher values of WC and WHtR. No significant differences in BMI between sexes were observed. Boys outperformed girls in the Maximal multistage 20-m shuttle run test (small effect) and the moderate ES for the estimated VO_2_max between boys and girls was obtained. According to the CDC reference percentiles in boys, the prevalence of general overweight/obesity using BMI was 14.7%, and the prevalence of abdominal overweight/obesity using WC and WHtR was 14.1 and 13.4%. In girls, the prevalence of general overweight/obesity using BMI was 14.5%, and the prevalence of abdominal overweight/obesity using WC and WHtR was 13.5 and 8.9%.

**Table 1:** Basic descriptive statistics of the study participants (*N* = 1,612).

	Total (*N* = 1,612)	Boys (*N* = 766)	Girls (*N* = 846)	Effect size	Value of *p*
	Mean (SD)	Mean (SD)	Mean (SD)		
Age (years)	9.7 (2.4)	9.8 (2.4)	9.6 (2.3)	0.08	0.148
Height (cm)	151.0 (17.6)	152.0 (19.4)	150.2 (15.7)	0.09	0.044
Weight (kg)	45.1 (19.1)	46.5 (13.3)	43.9 (14.0)	0.20	0.006
Body-mass index (kg/m^2^)	19.0 (3.5)	19.2 (3.7)	18.9 (3.3)	0.08	0.156
Waist circumference (cm)	65.4 (9.5)	67.1 (10.0)	64.0 (8.8)	0.31	<0.001
Waist-to-height ratio	0.43 (0.05)	0.44 (0.05)	0.43 (0.05)	0.20	<0.001
20-m shuttle run (level)	4.3 (1.9)	4.9 (2.2)	3.9 (1.4)	0.45	<0.001
VO_2_max (mlO_2_/kg/min)	47.0 (6.5)	50.7 (6.5)	43.6 (4.3)	1.09	<0.001

*p* < 0.05.

**Figure 1:**
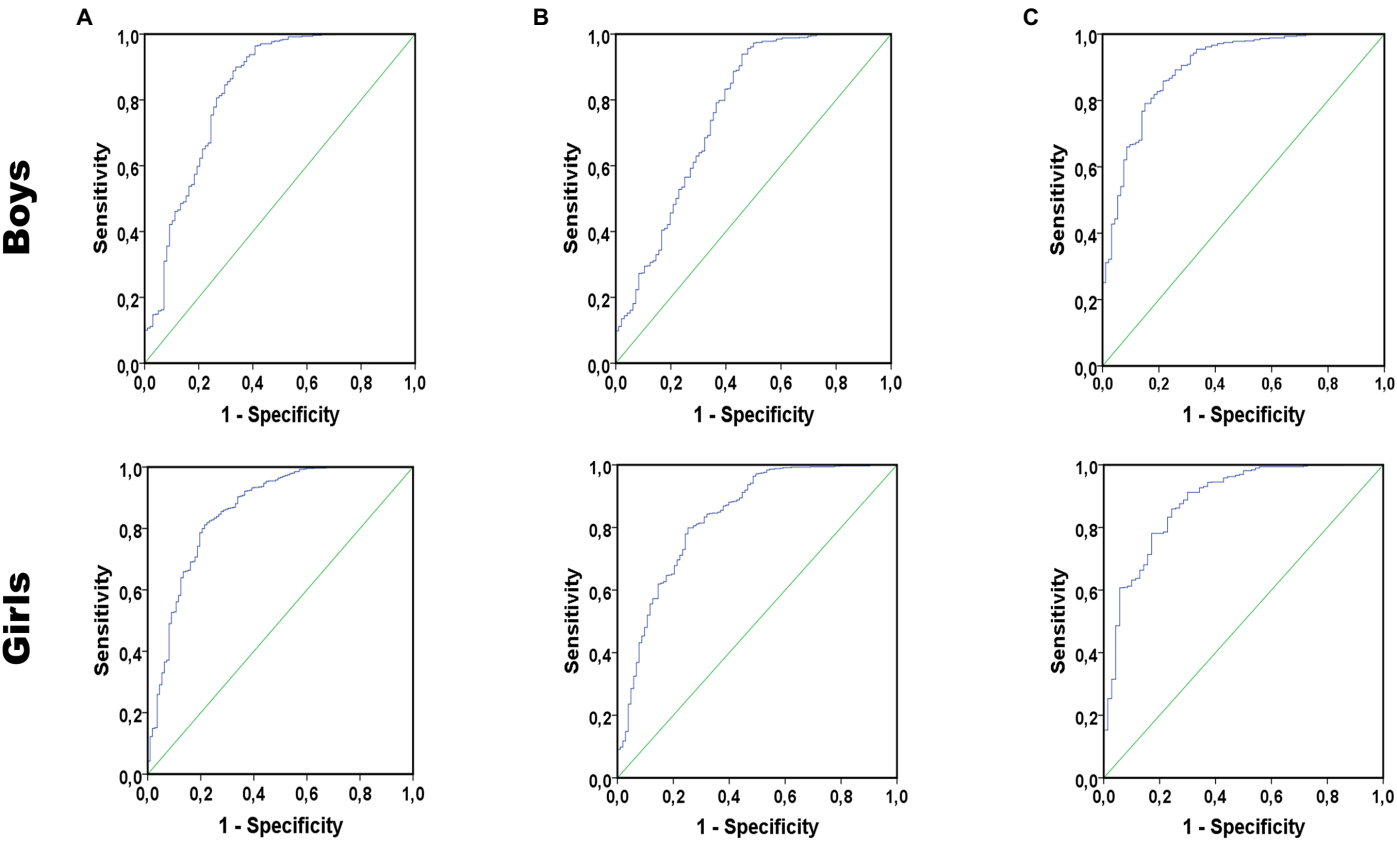
Receiver operating characteristic (ROC) curves of maximal oxygen uptake (VO_2_max) to detect overweight/obesity in boys and girls according to body-mass index (BMI; **A**), waist circumference (WC; **B**), and waist-to-height ratio (WHtR; **C**).

Figure 1 shows ROC curves of VO_2_max estimated by the Ruiz et al. (2008) equations to detect overweight/obesity in boys and girls according to BMI (A), WC (B), and WHtR (C). Diagnostic properties of VO_2_max to detect overweight/obesity in boys and girls are presented in Table 2. For both boys and girls, VO_2_max showed significant predictive capacity for overweight/obesity (AUCs > 0.75). The best VO_2_max cut-off points to detect overweight/obesity were observed for WHtR in both sexes, followed by BMI and WC. Boys with low CRF determined by the ROC were 17.04 (95% CI 10.39–27.93, *p* < 0.001), 10.52 (95% CI 6.50–17.01, *p* < 0.001), and 23.50 (95% CI 13.91–39.69, *p* < 0.001) more likely to be overweight/obese determined by BMI, WC, and WHtR. In girls, low CRF was associated with 14.89 (95% CI 9.12–24.31, *p* < 0.001), 11.25 (95% CI 6.94–18.22, *p* < 0.001), and 16.56 (95% CI 8.68–31.62) higher likelihood of being overweight/obese.

**Table 2:** Receiver operating curve cut-offs for cardiorespiratory fitness (CRF) to predict overweight/obesity for different anthropometric indices, stratified by sex.

Study variables	Body-mass index (kg/m^2^; ≥85^th^ percentile denotes overweight/obesity)				
VO_2_max (mlO_2_/kg/min)	AUC	95% CI	Std. error	Value of *p*	Cut-off point
Boys (*N* = 766)	0.83	0.78–0.88	0.027	<0.001	44.6
Girls (*N* = 866)	0.86	0.82–0.90	0.022	<0.001	41.0
	**Waist circumference (cm; ≥85^th^ percentile denotes overweight/obesity)**				
**VO_2_max (mlO_2_/kg/min)**	**AUC**	**95% CI**	**Std. error**	**Value of *p***	Cut-off point
Boys (*N* = 766)	0.77	0.71–0.83	0.031	<0.001	46.4
Girls (*N* = 866)	0.83	0.79–0.88	0.024	<0.001	40.8
	**Waist-to-height ratio (≥0.5 denotes overweight/obesity)**				
**VO_2_max (mlO_2_/kg/min)**	**AUC**	**95% CI**	**Std. error**	**Value of *p***	**Cut-off point**
Boys (*N* = 766)	0.90	0.86–0.93	0.019	<0.001	46.9
Girls (*N* = 866)	0.88	0.84–0.93	0.023	<0.001	40.7

Sensitivity, specificity, and Kappa statistics for newly developed cut-off points of VO_2_max are presented in Table 3. Sensitivity for detecting overweight/obesity according to different anthropometric indices was strong in both boys (>89%) and girls (>77%). Kappa statistics showed moderate correlations between CRF and overweight/obesity in both sexes. By using our newly proposed cut-off points for CRF, 16.7% of boys and 28.3% of girls had low CRF (*p* < 0.001).

**Table 3:** Sensitivity, specificity, and Kappa statistics for cardiorespiratory cut-offs and overweight/obesity in different anthropometric indices, stratified by gender.

Boys	Body-mass index (kg/m^2^; ≥85^th^ percentile denotes overweight/obesity)					
VO_2_max (mlO_2_/kg/min)	Normal^*^	Overweight/obesity	Chi-square test	Value of *p*	Kappa statistics	Value of *p*
≥44.6 mlO_2_/kg/min	90.8%	36.7%				
<44.6 mlO_2_/kg/min	9.2%	63.3%	177.8	<0.001	0.50	<0.001
**Girls**						
**VO_2_max (mlO_2_/kg/min)**						
≥41.0 mlO_2_/kg/min	80.2%	21.4%				
<41.0 mlO_2_/kg/min	19.8%	78.6%	163.5	<0.001	0.42	<0.001
**Boys**	**Waist circumference (cm; ≥85^th^ percentile denotes overweight/obesity)**					
**VO_2_max (mlO_2_/kg/min)**	**Normal**	**Overweight/obesity**	**Chi-square test**	**Value of *p***	**Kappa statistics**	**Value of *p***
≥46.4 mlO_2_/kg/min	89.5%	44.8%				
<46.4 mlO_2_/kg/min	10.5%	55.2%	118.5	<0.001	0.41	<0.001
**Girls**						
**VO_2_max (mlO_2_/kg/min)**						
≥40.8 mlO_2_/kg/min	79.2%	25.2%				
<40.8 mlO_2_/kg/min	20.8%	74.8%	128.2	<0.001	0.37	<0.001
**Boys**	**Waist-to-height ratio (≥0.5 denotes overweight/obesity)**					
**VO_2_max (mlO_2_/kg/min)**	**Normal**	**Overweight/obesity**	**Chi-square test**	**Value of *p***	**Kappa statistics**	**Value of *p***
≥46.9 mlO_2_/kg/min	91.4%	31.2%				
<46.9 mlO_2_/kg/min	8.6%	68.8%	209.3	<0.001	0.55	<0.001
**Girls**						
**VO_2_max (mlO_2_/kg/min)**						
≥40.7 mlO_2_/kg/min	77.4%	17.1%				
<40.7 mlO_2_/kg/min	22.6%	82.9%	114.3	<0.001	0.31	<0.001

* denotes using percentages (%).

## Discussion

The main purpose of the study was to define optimal criterion-referenced cut-points for CRF associated with overweight/obesity, according to BMI, WC, and WHtR. Our main findings are: (1) estimated CRF accurately predicts the presence of overweight/obesity in both boys and girls; (2) the optimal cut-off points for CRF are between 44.6 and 46.9 mlO_2_/kg/min in boys and between 40.7 and 41.0 mlO_2_/kg/min in girls; and (3) a strong sensitivity and moderate correlations between CRF and overweight/obesity are found in both sexes.

Health-related criterion-referenced standards for CRF have been published previously (Mesa et al., 2006; Ruiz et al., 2007, 2015; Lobelo et al., 2009; Adegboye et al., 2011; Moreira et al., 2011; Welk et al., 2011; Boddy et al., 2012; Silva et al., 2012, 2016, 2018; Lang et al., 2019). Most of the studies have used the Maximal multistage 20-m shuttle run test to assess the level of CRF. Of these, only a handful of studies have examined cut-off points for CRF to define overweight/obesity (Ruiz et al., 2007, 2015; Lobelo et al., 2009; Adegboye et al., 2011; Moreira et al., 2011; Boddy et al., 2012; Silva et al., 2012, 2018). The AUC in these studies ranged between 0.54 and 0.83, indicating a great heterogeneity of the reported criterion-referenced standards for CRF (Lang et al., 2019). Our findings of 44.6–46.9 mlO_2_/kg/min in boys and 40.7–41.0 mlO_2_/kg/min in girls are in line with previous sex- and age-specific studies (Boddy et al., 2012; Silva et al., 2012, 2018). Specifically, a study by Boddy et al. (2012) showed cut-off points for CRF of 46.6 and 41.9 mlO_2_/kg/min for boys and girls, when criteria were BMI and WC. Similarly, Silva et al. (2012) obtained the results, where the best discriminatory cut-off points were between 43.1 and 46.3 mlO_2_/kg/min in boys and between 32.6 and 43.9 mlO_2_/kg/min in girls. Recently, a population-based study conducted among 8,740 Canadian children aged 8–12 years showed, that the optimal cut-off points for VO_2_max estimated using the Léger et al. (1984) to detect obesity by BMI and WC were 43.9 and 43.4 mlO_2_/kg/min in boys and 43.0 and 42.1 mlO_2_/kg/min in girls.

Although cut-off points of CRF are similar between the studies, it should be noted, that different equations have been proposed to estimate VO_2_max from the Maximal multistage 20-m shuttle run test (Léger et al., 1984; Ruiz et al., 2008; Burns et al., 2015). Although the Ruiz et al. (2008) equation was used to estimate VO_2_max in this study, previous evidence has suggested not considering body fat or physical growth indicators (height and weight), because of the risk of collinearity (Cureton and Mahar, 2014). However, the problem of using past equations (developed in the 20th century) is the nature of CRF and obesity, where the most recent study conducted among Croatian youth has shown a decrease in CRF and an increase in overweight/obesity from 1999 to 2014 (Kasović et al., 2021b). Thus, previous equations may not be feasible to estimate VO_2_max nowadays.

It has been well-documented, that physical fitness represents a powerful marker of health during childhood and adolescence period (Ortega et al., 2008). Since physical fitness tracks moderately to highly well from childhood to adulthood (Shigaki et al., 2020), special interventions and policies aiming to target “a risky” group of children with low CRF to detect those with overweight/obesity should be a high priority in a school-based setting.

## Limitations

This study is not without limitations. First, the nature of the study design (cross-sectional) cannot determine the causality of the association between CRF and anthropometric indices. Second, it is speculated that more physically active children would be more involved and motivated in the study of such purpose. Thus, potential selection bias cannot be excluded. Third, the Maximal multistage 20-m shuttle run test was used to estimate VO_2_max. Although this test has been widely used in children (Ortega et al., 2008, 2011), more direct measures of VO_2_max (treadmill or bicycle ergometers) might have given different cut-off points for CRF. Fourth, BMI, WC, and WHtR were used as a proxy of general and abdominal overweight/obesity. Although benefits of the aforementioned anthropometric indices have been highlighted, more sophisticated tools, like dual X-ray absorptiometry should be used in future research to assess the level of body composition in school–aged children.

## Conclusion

It could be concluded that VO_2_max estimated from the Maximal multistage 20-m shuttle run test (using sex, age, weight, height, and stage completed) accurately identified overweight/obesity in Croatian children aged 7–14 years. VO_2_max estimated by the Ruiz et al. (2008) equation showed good discriminative ability for overweight/obesity. Our newly proposed cut-off points for CRF should be implemented in the school context as a screening tool for overweight/obesity in primary school children. Moreover, such cut-off points should be compared to other countries and in different populations, to establish sex- and age-specific criterion-referenced standards for health-related purposes.

## Data Availability Statement

The original contributions presented in the study are included in the article/supplementary material, further inquiries can be directed to the corresponding author.

## Ethics Statement

The studies involving human participants were reviewed and approved by Faculty of Kinesiology, University of Zagreb, Croatia. Written informed consent to participate in this study was provided by the participants’ legal guardian/next of kin.

## Author Contributions

MK, LŠ, and VP designed the study and collected and analyzed the data. MK, LŠ, VP, VŠ, and IB contributed to interpretation of the data, drafting, and revising the manuscript. All authors contributed to the article and approved the submitted version.

## Conflict of Interest

The authors declare that the research was conducted in the absence of any commercial or financial relationships that could be construed as a potential conflict of interest.

## Publisher’s Note

All claims expressed in this article are solely those of the authors and do not necessarily represent those of their affiliated organizations, or those of the publisher, the editors and the reviewers. Any product that may be evaluated in this article, or claim that may be made by its manufacturer, is not guaranteed or endorsed by the publisher.
